# The left ventricular outflow tract and carotid artery velocity time integrals

**DOI:** 10.3389/fmedt.2024.1320810

**Published:** 2024-01-24

**Authors:** Jon-Emile S. Kenny

**Affiliations:** ^1^ Health Sciences North Research Institute, Sudbury, ON, Canada; ^2^ Flosonics Medical, Toronto, ON, Canada

**Keywords:** fluid responsiveness, carotid artery, Doppler ultrasound, corrected flow time, measurement error, velocity time integral, left ventricular outflow tract (LVOT), shock

## Abstract

The left ventricular outflow tract velocity time integral (LVOT VTI) is commonly used in the intensive care unit as a measure of stroke volume (SV) and how the SV changes in response to an intervention; therefore, the LVOT VTI is used to guide intravenous fluid management. Various peripheral Doppler surrogates are proposed to infer the LVOT VTI (e.g., measures from the common carotid artery). A recently-described, novel method of insonation has an excellent ability to detect change in the LVOT VTI. This approach raises important facets of Doppler flow and insonation error, as well as the general principles at play when using a peripheral artery to infer changes from the left ventricle. Relating the VTI of a peripheral artery to the LVOT VTI was recently described mathematically and may help clinicians think about the Doppler relationship between central and peripheral flow.

## Background

Both observational studies (1, 2) and a randomized trial (3) demonstrate that when intravenous (IV) fluid is administered based on stroke volume change (SV_Δ_) in sepsis and septic shock, patient-centered outcomes improve. Measuring SV_Δ_, however, is challenging. One ultrasound-based approach is to use the change in left ventricular outflow tract velocity time integral (LVOT VTI_Δ_) as a surrogate for SV_Δ_ (4). Though LVOT VTI_Δ_ is an accurate way to assess SV_Δ_ (5, 6), obtaining and maintaining a stable ultrasound window of the LVOT can be limiting. This is especially true when the patient has large body habitus, is in a position other than supine or semi-recumbent (e.g., prone), has lung hyperinflation (e.g., ventilated with high expiratory pressure, lung disease that causes air-trapping such as asthma), or if there is a physical barrier to the thorax (e.g., in the operating room). Therefore, clinicians have sought large peripheral vessels (e.g., the carotid artery) that can be easier to assess with Doppler ultrasonography. To this end, a recently-described, novel method of insonating the common carotid artery was proposed as an LVOT VTI surrogate. As originally described by Cheong and colleagues, this approach entails using a cardiac probe placed in the supraclavicular fossa to “look down” at the left common carotid artery from its bifurcation off of the aortic arch such that the insonation angle is zero (7, 8).

Most recently, this group has reported data from 50 critically-ill patients who were dichotomized into fluid responders and non-responders based upon a +15% change in LVOT VTI (8). To investigate whether the carotid artery velocity time integral (VTI_CA_) could be used to differentiate volume responders and non-responders, the percent change in the VTI_CA_ before and during a passive leg raise (PLR) was calculated using the aforementioned, unique approach (7). The mix of patients enrolled reflected a typical distribution of patients in a medical intensive care unit (e.g., 46% were intubated, 34% had left heart dysfunction, 10% had right heart dysfunction, 52% were on vasopressors). An 11% augmentation of VTI_CA_ was 77% sensitive and 79% specific for identifying fluid responders from non-responders (i.e., area under the receiver operator curve of 0.87), which is an excellent diagnostic accuracy in this patient population. These data bring 3 broad issues to the fore: (1) their results as compared to previously-published studies on this topic and how this relates to sources of insonation error, (2) the relationship between changing VTI_CA_ and the corrected flow time of the carotid artery (i.e., ccFT) and (3) the mechanism by which changes in the LVOT VTI are reflected in the VTI_CA_.

## Previous literature and sources of error

First, as mentioned by the authors, there is controversy in this space (9). Initially, Marik and colleagues found that a 20% increase in carotid artery flow had superb diagnostic characteristics for differentiating a +10% rise in SV (10) and this was echoed by an investigation by Effat et al. (11). However, disappointing results by Girotto et al. (12) as well as Abbasi and colleagues (13) and, more recently, Patnaik et al. (14) have followed. Importantly, all of these authors employed change in total carotid artery flow as the SV or cardiac output surrogate of choice. Carotid artery flow requires carotid artery diameter measurement both before and during the preload challenge such that changes in vascular area are captured. An important consideration here is that a small error in diameter measurement leads to exponentially-enlarged flow error. For example, in a 6-millimeter (mm) carotid artery, a 1 mm error in diameter translates to a 30% flow error, which is clinically-unacceptable. Therefore, error in carotid artery area calculation could be at fault for some of these discrepancies. If only VTI_CA_ is considered, the area error is moot, however, insonation angle error remains a problem (15). Using the change in VTI_CA_ to predict preload responsiveness in critically-ill patients was also studied by Chowhan and colleagues with LVOT VTI as the reference standard (16). In septic patients without shock, the area under the receiver operating curve (AUROC) for the ability of VTI_CA_ to detect change in LVOT VTI was 0.90, which is excellent. However, in their septic shock group, the AUROC was only 0.69 (17). Nevertheless, in their report VTI_CA_ was captured using the traditional method of insonating the carotid artery in the neck with a linear probe at 60 degrees insonation angle. At this angle, a 5-degree misjudgement leads to a nearly 20% flow error, which is also clinically-unacceptable.

## The corrected flow time

Second, it has been argued that mitigating the insonation angle error can be accomplished by using the corrected flow time of the carotid artery (ccFT) as a surrogate for SV_Δ_ (18–21). Cheong and colleagues appropriately point out that in one such study—which employed a model of moderate-to-severe hemorrhage in healthy volunteers with uncalibrated pulse contour analysis as the gold standard—was limited by the small number of participants (19). To address this criticism, the authors studied an additional 14 healthy volunteers for the same paradigm and added aortic VTI as another reference standard (22). Again, these authors found a strong, linear correlation between carotid Doppler measures and ascending aortic VTI as measured by the USCOM device. This group has also observed a strong, linear correlation between changing ccFT cand VTI_CA_, thus these measures are likely physiologically-linked (23). As mentioned by Cheong and colleagues, composite measures integrating both ccFT and VTI_CA_ are of great interest.

## Relating central-to-peripheral velocity time integrals

Third, the relationship between changing LVOT VTI and VTI_CA_ deserves brief elaboration. In response to their earlier publication (7), the following Equation 1 was derived to relate LVOT VTI to VTI_CA_ (24):

(1) $$\;\mathrm{VT}I_{\mathrm{CA}}=K\times \left[\;\frac{\mathrm{CS}A_{\mathrm{LVOT}}}{\mathrm{CS}A_{\mathrm{CA}}}\;\times \;CA_{\mathrm{FLOWFRAC}}\;\times \;\mathrm{VT}I_{\mathrm{LVOT}}\right]\;\;$$

Here, the VTI_CA_ is the product of the flow profile in the carotid artery (K), the ratio of LVOT cross sectional area (CSA) to carotid artery CSA, the fraction of flow to one carotid artery [i.e., CA_flowfrac_, normally about 0.10 (25)] and the LVOT VTI. Given this equation, a +15% increase in LVOT VTI should translate to a +15% in the VTI_CA_, but only if all other variables are unchanged. Yet, the optimal VTI_CA_ threshold found by Cheong and colleagues is +11%. Thus, one (or more) of the variables in the equation fell in value during the PLR, driving down the optimal VTI_CA_ threshold. The most plausible explanation is increased CA cross-sectional area (assuming the LVOT CSA is unchanged) (26). Indeed, the responders had a statistically and clinically significant increase in mean arterial pressure during the PLR; increased pressure in a central, elastic artery like the common carotid likely increased arterial diameter (27). With increased CA CSA it is also possible that the flow profile becomes more plug-like which would reduce K and, therefore, VTI_CA_ as well. Thus, increased CA CSA reduces VTI_CA_ relative to LVOT VTI and produces false negatives. But some false positives were also observed. Why might the VTI_CA_ increase disproportionately in non-responders? Again, from the equation above an increase in either K, the ratio of LVOT to CA CSA or flow fraction would yield this result. The most conceivable explanation is increased flow fraction towards the carotid artery relative to the body (i.e., CA_flowfrac_). During the PLR, this is possible simply by gravitational changes. Moving from head above the heart, to supine with the legs elevated could make common carotid artery flow more favourable. Furthermore, norepinephrine preferentially directs blood to the brain (28); this medication may have had additive effects with gravitational gradient changes during PLR.

In conclusion, Cheong and colleagues elegantly circumvent some aspects of human measurement variability, especially regarding vascular cross-sectional area and insonation angle. Further investigation might include integrating their approach into a protocol for IV fluid provision with focus on patient-centred outcomes (29).

## Data Availability

The original contributions presented in the study are included in the article/Supplementary Material, further inquiries can be directed to the corresponding author.
